# The hazards of lack of co-registration of ictal brain SPECT with MRI: A case report of sinusitis mimicking a brainstem seizure focus

**DOI:** 10.1186/1471-2385-4-2

**Published:** 2004-11-29

**Authors:** Tracy Butler, Lawrence J Hirsch, Jan Claassen

**Affiliations:** 1 Functional Neuroimaging Laboratory, Weill Medical College of Cornell University, New York, NY, USA; 2 Columbia Comprehensive Epilepsy Center, Columbia University College of Physicians and Surgeons, New York, NY, USA; 3 Columbia Comprehensive Epilepsy Center, Columbia University College of Physicians and Surgeons, New York, NY, USA

## Abstract

**Background:**

Single photon emission computed tomography (SPECT) following injection of radiotracer during a seizure is known as ictal SPECT. Comparison of an ictal SPECT study to a baseline or interictal study can aid identification of a seizure focus.

**Case presentation:**

A young woman with encephalitis and refractory seizures underwent brain SPECT during a period of frequent seizure-like episodes, and during a seizure-free period. A focal area of increased radiotracer uptake present only when she was experiencing frequent seizure-like episodes was originally localized to the brainstem, but with later computerized co-registration of SPECT to MRI, was found to lie outside the brain, in the region of the sphenoid sinus.

**Conclusion:**

Low-resolution SPECT images present difficulties in interpretation, which can be overcome through co-registration to higher-resolution structural images.

## Background

Radiotracers used for brain single photon emitted computed tomography (SPECT) pass the blood-brain barrier and bind intracellularly on their first pass through the circulation, providing a "snapshot" of cerebral perfusion at a particular timepoint. When injected during a focal epileptic seizure, an area of significantly increased radiotracer uptake typically corresponds to the region of maximal abnormal activity, often the seizure focus. This ictal pattern of cerebral blood flow can be compared to an interictal/baseline pattern obtained when the patient is not having a seizure, to provide unique information about the nature and location of a patient's epileptic focus, which can be used to guide therapy [See [1] for a review of the use of SPECT in epilepsy].

## Case presentation

**Figure 1:**
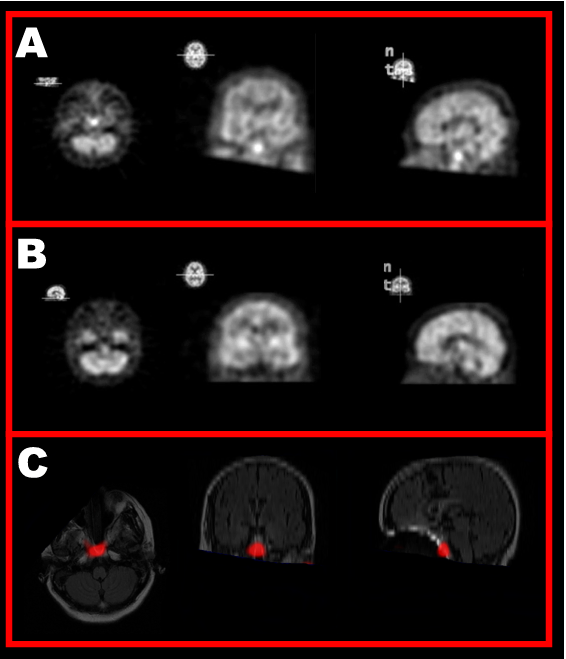
A: Axial, coronal and sagittal 99mTc-HMPAO SPECT images of the brain obtained during a facial twitching episode show moderate to severe heterogeneous cerebral hypoperfusion with an apparent focus of increased radiotracer uptake near the midbrain. B: 99mTc-ECD SPECT image of the brain obtained two weeks after (A) during a period free from twitching shows resolution of the area of increased uptake. C: Computer-generated depiction of A-B overlayed onto a co-registered MRI shows the area of increased uptake to lie outside the brain. See [5] or  for image analysis and alignment methodology.

A previously-healthy young woman developed behavioral changes followed by seizures and refractory status epilepticus. She was diagnosed with encephalitis and treated with antiviral and multiple antiepileptic agents. She required nasotracheal intubation and mechanical ventilation for respiratory support. She experienced persistent episodes of facial twitching resembling seizures. These episodes were not however associated with an ictal EEG pattern on continuous video/EEG monitoring. To clarify the nature of these episodes, 99mTc-HMPAO was injected during a period of frequent twitching. Brain SPECT showed a prominent focus of increased uptake interpreted by the radiologists and clinical team as arising in the upper brainstem (Figure 1A.) A repeat study (using 99mTc-ECD) two weeks later, when twitching was no longer occurring, showed resolution of this increased uptake (Figure 1B). These findings were considered to support a diagnosis of seizures/repetitive myoclonus originating from the brainstem. Although brainstem seizures in humans remain a controversial entity, there are reports in the medical literature of seizures and seizure-like movements related to brainstem lesions [2-4] making this a plausible diagnosis in this case, based on clinical, EEG and SPECT findings. Antiepileptic and supportive treatment were continued, but the patient's disease proved fatal. No autopsy was performed.

With later co-registration of SPECT data to the patient's MRI using a computerized medical image registration and visualization program, [5] the area of increased radiotracer uptake was seen to be anterior to the brain, likely in the sphenoid sinus, consistent with sinusitis (Figure 1C). MRI showed prominent maxillary and sphenoidal sinus mucosal thickening also consistent with sinusitis. Chronic nasotracheal intubation is commonly accompanied by sinusitis, [6] which may resolve with removal of the offending tube, as was performed in this case when the patient underwent tracheotomy during the interval between the first and second SPECT scans.

## Conclusions

The nature of this patient's twitching episodes remains uncertain, though they were likely due to epileptic activity involving too small a volume of brain to be detectable by either EEG or SPECT. Her case is described here to illustrate a previously-unreported pitfall in the interpretation of brain SPECT studies, with the goal of emphasizing the importance of image co-registration in nuclear medicine. Diagnostic limitations inherent to low-resolution images can be overcome through use of computer software to accurately co-register brain images of different modalities acquired separately (as described here) or hybrid medical devices such as PET-CT scanners to automatically fuse simultaneously-acquired images. Both methods have been shown to be superior to visual inspection and mental integration of information by experienced radiologists [7,8].

## List of abbreviations

SPECT: single photon emitted computed tomography; MRI: magnetic resonance imaging; EEG: electroencephalography

## Competing interests

The author(s) declare that they have no competing interests.

## Authors' contributions

TB performed image analysis and co-registration and drafted the manuscript. LJH identified the patient, conceived of the study, and drafted a preliminary version of the manuscript. JC participated in data collection and analysis. All authors read and approved the final manuscript.

## Pre-publication history

The pre-publication history for this paper can be accessed here:


